# Knee effusion evaluated by ultrasonography warns knee osteoarthritis patients to develop their muscle atrophy: a three-year cohort study

**DOI:** 10.1038/s41598-020-65368-4

**Published:** 2020-05-21

**Authors:** Daisuke Chiba, Seiya Ota, Eiji Sasaki, Eiichi Tsuda, Shigeyuki Nakaji, Yasuyuki Ishibashi

**Affiliations:** 1 Department of Orthopaedic Surgery, Hirosaki University Graduate School of Medicine, Hirosaki, Japan; 2 Department of Rehabilitation Medicine, Hirosaki University Graduate School of Medicine, Hirosaki, Japan; 3 Department of Social Medicine, Hirosaki University Graduate School of Medicine, Hirosaki, Japan

**Keywords:** Skeletal muscle, Osteoarthritis

## Abstract

This study aimed to elucidate the relationship between the quantitative value of suprapatellar effusion and the longitudinal changes in lower-extremity muscle mass (MM) in a cohort with knee osteoarthritis (KOA). Fifty-three subjects (106 legs) with bilateral radiographic KOA at baseline (BL) were enrolled. MM was calculated by bioimpedance analysis three times at BL, and at the one-year (1Y) and three-year (3Y) follow-ups. The longitudinal change in the lower-extremity MM was calculated by subtracting MM_BL_ from MM_1Y_, and MM_1Y_ from MM_3Y_. Subjects with ≥1.0 z-score loss were defined as having severe MM loss (SMML). Effusion was evaluated as the sagittal area of suprapatellar pouch (mm^2^) by ultrasonography. The ROC curve was drawn to determine the cut-off of effusion area. General estimating equations (GEEs) were conducted with the prevalence of SMML as the dependent variable and with the cut-off of effusion area as the independent variable. Sixteen legs (15.1%) demonstrated SMML_BL-1Y_ and another sixteen legs demonstrated SMML_1Y-3Y_. GEEs revealed that individuals with ≥90 mm^2^ effusion had significantly higher odds of SMML_BL-1Y_ prevalence (Odds ratio: 21.561; P-value: 0.003). Individuals with leachate knee effusion at BL had a significant risk of losing MM through the first year of the initial knee effusion assessment.

## Introduction

Knee osteoarthritis (KOA) is a common form of arthritis. KOA degrades not only cartilage tissue but also other tissue such as bone, synovium, and ligament tissues^1^. The muscular system surrounding the knee joint is consistently deteriorated with the incidence or development of KOA. For instance, studies show that quadriceps strength is weakened in people diagnosed with KOA^2,3^ as well as in those with severe knee pain, stiffness, and dysfunction^4^. Accordingly, weakness of quadriceps strength has been associated not only with worsening knee pain or function^5–7^, but also with the development of osteoarthritic modifications^7–11^. However, there is limited evidence regarding how KOA pathology affects changes in the muscle mass (MM) of affected lower extremities in comparison with changes in muscle strength.

Knee joint effusion is a characteristic pathological finding in patients with KOA. Effusion is associated with the severity of synovitis in the osteoarthritic knee joint, and with generating both knee symptoms and disease progression^12^. In particular, knee effusion has been reported to inhibit muscle contraction of the quadriceps and has been associated with weakness of quadriceps strength^13–24^. Unfortunately, in patients with KOA, there is a lack of evidence associating pathological mechanisms with the severity of knee effusion and MM loss of the lower-extremities. In the present cohort study, there were two purposes: first, based on a previously reported ultrasonographic technique^25^, we retrospectively determined the quantitative value of knee effusion in patients with KOA at baseline (BL) to identify those who demonstrated loss of lower-extremity MM; second, we analyzed the relationship between the quantitative value of knee effusion at BL and changes in lower-extremity MM for the cohort. Our hypothesis was that those who demonstrated more leachate knee effusion would be more likely to develop the loss of lower-extremity MM during the 3-year follow-up.

## Materials and Methods

### The iwaki health promotion project

The Iwaki Health Promotion Project is a community-based program that promotes improvement of the average life span. This program was initiated in 2005. Approximately, 1000 adults aged ≥20 years who live in the Iwaki area of Hirosaki City, Japan (the western Aomori prefecture) participate in this program annually. In addition to an orthopedic specialist, physicians, general surgeons, gynecologists, urologists, psychiatrists, dermatologists, and dentists are involved in this project. Our department collects biochemical and biomechanical data related to knee OA, including that of the spine, hip, and other joints.

### Study subjects

Overall, 1167 participants underwent the health check-up in 2014 (BL). Of these participants, the current study used the same data set from our previous report^25^. Initially, 127 volunteers diagnosed with radiographic OA of both knees and with data relative to ultrasonographic examination at BL were enrolled. Radiographic KOA was defined by the Kellgren-Lawrence grade (KLG); both knees of each participant presented a KLG of ≥2. Participants had no history of hepatic or renal disease, malignancy, or rheumatoid arthritis. Furthermore, the included participants had no history of severe knee trauma or knee surgery, nor were taking any analgesic medications at BL (e.g., non-steroidal anti-inflammatory drugs, acetaminophen, tramadol, etc.) prescribed by a medical institution. All methods were performed in accordance with the relevant guidelines and regulations. The Ethics Committee of the Hirosaki University Graduate School of Medicine approved the present study, and all subjects provided written informed consent before participation. Finally, 53 subjects (41.7%), who had joined the health check-up program and completed the measurement of MM in both 2015 (one-year follow-up: 1Y) and in 2017 (three-year follow-up: 3Y), were eligible for the current study.

### Questionnaire of knee symptoms and exercise

All subjects completed the Knee Injury and Osteoarthritis Outcome Scale (KOOS) questionnaire individually at the BL to assess the severity of knee pain. If needed, staff members of our department assisted elderly subjects who had difficulty reading the questionnaire due to presbyopia, for instance. The KOOS is a 42-item, knee-specific, self-administered questionnaire with five subscales: pain, symptom, activities of daily living (ADL), sport and recreation (sports), and knee-related quality of life (QOL). All items were scored from 0–4 and then summed. Then, the raw scores were transformed to a 0–100 scale, in which 100 represented an absence of a problem and 0 represented the most extreme problem. A separate score was calculated for each of the five subscales. The KOOS score is a sufficiently reliable, valid, and responsive tool for assessing pain or stiffness and other symptoms, including ADL, function in sports and recreation, and QOL associated with many types of knee disorders^26,27^. In the current study, the severity of knee pain at the BL was evaluated by the KOOS Pain scale. The frequency of exercise was also recorded via a questionnaire that asked how many days they performed any kind of exercise in a week. Participants who performed exercise more than once per week were defined as positive for exercise habit on a binary scale.

### Evaluation of lower-extremity muscle mass by bioimpedance analysis

The MM of each lower extremity was evaluated separately by bioimpedance analysis (BIA) using a multifrequency body composition analyzer (TANITA MC-190, Tanita Corp., Tokyo, Japan)^28–32^ on three occasions: BL and at 1Y and 3Y follow-up. The reliability of the BIA measurement has been validated (correlation coefficient > 0.9) using dual X-ray absorptiometry to measure MM^28–30^ and utilized in large Japanese population studies to evaluate MM^31,32^.

In this study, the MM was measured and analyzed using the value obtained for each lower extremity. The degree of change was calculated from BL to 1Y (ΔMM_BL-1Y_ = [MM_1Y_] − [MM_BL_]) and from 1Y to 3Y (ΔMM_1Y-3Y_ = [MM_3Y_]64919 − [MM_1Y_]). Specifically, subjects showing a decline in MM of −1.0 z-score of ΔMM over a certain follow-up period were defined as having severe MM loss (SMML).

### Quantitative evaluation of suprapatellar effusion by ultrasonography

**Figure 1:**
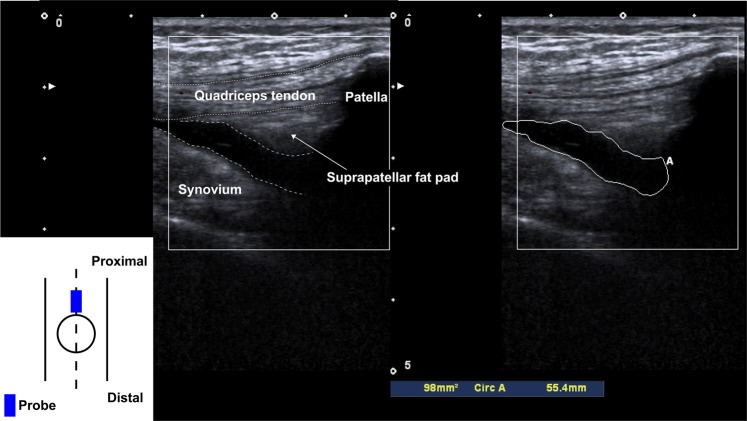
Quantitative evaluation of the suprapatellar effusion area by ultrasonography The ultrasound probe is longitudinally placed at the center at the proximal pole of the patella. Based on the acquired sagittal image, the boundary of echo-free space, which corresponds to the suprapatellar pouch, is traced and the area of suprapatellar effusion (mm^2^) is automatically calculated. Synovial tissue and suprapatellar fat pad are excluded from the current measurement area.

According to our previously described study^25^, suprapatellar effusion was quantitatively evaluated by the sagittal area using ultrasonography. During the examination, the subjects were on a bed in a supine position with both knees semi-flexed. A pillow was put underneath both knees without any quadriceps contraction. The ultrasound probe (12-MHz, Viamo^TM^; Canon Medical Systems Corp., Otawara, Japan) was placed longitudinally at the center of the proximal pole of the patella (Fig. 1). On the acquired sagittal image, the boundary of echo-free space, which corresponded to the suprapatellar pouch, was traced and the area of suprapatellar effusion (mm^2^) was automatically calculated. Synovial tissue and suprapatellar fat pads were excluded from the measurement area (Fig. 1).

### Statistical analysis

Statistical tests were conducted using SPSS v 24.0 (SPSS Inc., Chicago, IL, USA). To determine the cut-off value of the suprapatellar effusion area to detect the risk of SMML, the receiver operating characteristic (ROC) curve was drawn and the Youden index (YI) (YI = [Sensitivity] + [Specificity] − 1) was calculated. Based on the higher YI, two cut-off values were determined with one demonstrating higher specificity and the other demonstrating higher sensitivity. At BL, all subjects were divided into three groups according to two cut-off values of effusion area. Continuous values among these three groups on each occasions were compared by mixed linear model; categorical values were compared by generalized estimating equations (GEEs). In these models, the subjects were treated as random effect variables.

The MM values at the three time points (BL, 1Y, and 3Y) were compared by repeated measured ANOVA (post-hoc test: Bonferroni). Finally, according to the cut-off values of effusion area, univariate and multivariate GEEs were conducted to evaluate the relationship between severity of knee effusion and the prevalence of SMML in the affected lower-extremity. Consistently, in the repeated measures ANOVA and GEEs, the subjects were also treated as random effect variables.

Single linear regression was conducted with the ΔMM as the dependent variable, and with the continuous value of effusion area at BL as the independent variable. Single logistic regression was conducted with the prevalence of SMML as the dependent variable, and with the cut-off of effusion area as the independent variable. Multiple regressions were conducted by including the BL demographic variables, such as age, sex, body mass index (BMI), MM, KOOS pain scale, and exercise habit as confounding variables. Statistical significance was defined if the P-value was ≤0.05.

### Ethics approval and consent to participate

The Ethics Committee of the Hirosaki University Graduate School of Medicine approved the present study.

### Consent for publication

All subjects provided written informed consent before participation.

## Results

Overall, MM_BL_ (6.29 ± 1.14 kg) was higher than MM_3Y_ (6.20 ± 1.18 kg; P < 0.001, repeated measures ANOVA) and MM_1Y_ (6.24 ± 1.12 kg) was higher than MM_3Y_ (P = 0.002, repeated measures ANOVA). Based on the −1.0 z-score of ΔMM, a decline of 0.40 kg MM or more was defined as SMML_BL-1Y_ and 0.43 kg MM or more was SMML_1Y-3Y_. Of the 106 legs from the 53 participants included in this study, the prevalence of SMML_BL-1Y_ and SMML_1Y-3Y_ was 15.1% (16 legs), respectively. There were no legs that demonstrated a decline in MM of −1.0 Z-score of ΔMM during both BL-1Y and 1Y-3Y.

**Figure 2:**
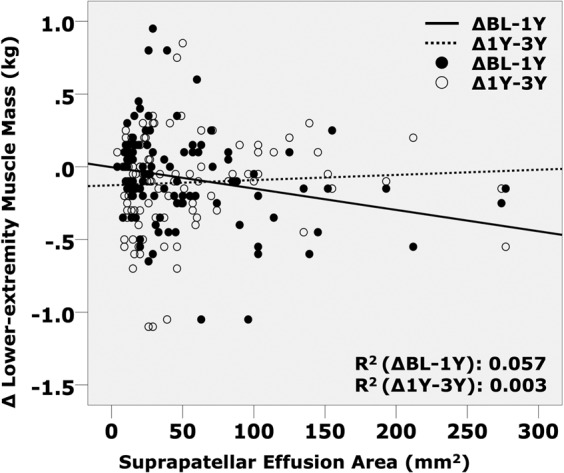
Scatter graph of suprapatellar effusion and change in lower-extremity muscle mass. BL: Baseline; 1Y: one-year follow-up; 3Y: three-year follow-up. ΔBL-1Y is calculated by subtracting the muscle mass (MM) of baseline from one-year and Δ1Y-3Y by subtracting MM at the 1Y-follow-up from that at the 3Y-follow-up.

Based on the scatter graph comparing effusion area and change (Δ) in lower-extremity MM, the suprapatellar effusion area was negatively correlated with ΔMM_BL-1Y_ (R^2^ = 0.057; P = 0.014). Conversely, this effusion area was positively correlated with ΔMM_1Y-3Y_; the slope of the approximate line was gradual in comparison to that of ΔMM_BL-1Y_ (R^2^ = 0.003; P = 0.557, Fig. 2).

**Figure 3:**
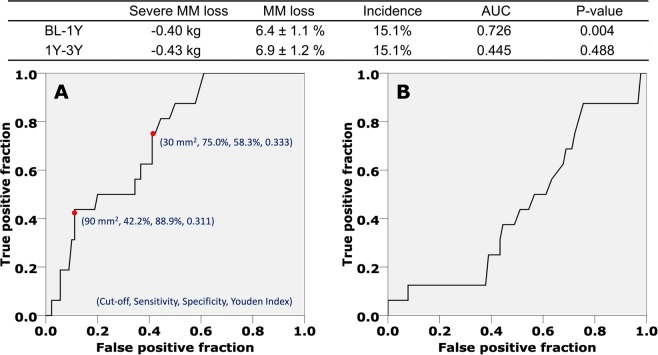
The receiver operating characteristic curve defining the suprapatellar effusion cut-off value to predict the risk of severe muscle mass loss in the lower extremity (**A**) from baseline to the one-year follow-up (**B**) from the one-year follow up to the three-year follow-up.

Based on the ROC curve for the cut-off of suprapatellar effusion at BL, 30 mm^2^ demonstrated both higher YI and sensitivity (0.333 and 75.0%, respectively) and 90 mm^2^ demonstrated both higher YI and specificity (0.311 and 88.9%, respectively). These two cut-off values were statistically significant for predicting risk of subjects exhibiting SMML_BL-1Y_ (Fig. 3A). On the contrary, the suprapatellar effusion area was not a significant predictor of SMML_1Y-3Y_ risk (Fig. 3B). According to the two cut-off values, the subjects were divided into three groups; (A): having <30 mm^2^ effusion area; (B): having 30≤ effusion area <90 mm^2^; (C): having ≥90 mm^2^ effusion area.

In the group (A), the MM_BL_ and MM_1Y_ were significantly higher than the MM_3Y_. In the group (B), the MM_BL_ was higher than the MM_1Y_ and MM_3Y_; the MM_1Y_ was also higher than the MM_3Y_. In the group (C), the MM_BL_ was higher than the MM_1Y_ and MM_3Y_ (Table 1). Cross-sectionally, the mean MM values were not different among three groups on each three occasions. At the BL, the mean KOOS Pain scale was significantly lower in the group (C) than the group (A) (Table 1). The prevalence of KLG 2 was higher in the group (A); that of KLG 4 was higher in the group (B); that of KLG 3 was higher in the group (C) than the other groups (Table 1).

**Table 1:** Demographic data from baseline to the three-year follow-up based on the cut-off of suprapatellar effusion area.

	(A) Effusion area <30 mm^2^ (N = 56)	(B) 30≤ Effusion area <90 mm^2^ (N = 33)	(C) Effusion area ≥90 mm^2^ (N = 17)
Effusion area^a^ (mm^2^)	17.0 ± 6.5	53.8 ± 16.3^*^	148.0 ± 58.7^*,†^
Age^a^	66.8 ± 8.1	67.3 ± 10.8	71.1 ± 8.6
BMI^a^ (kg/m^2^)	23.1 ± 2.8	23.7 ± 3.8	24.0 ± 2.8
Male/Female^a^	9/47 (16.1/83.9%)	11/22 (33.3/66.7%)	6/11 (35.3/64.7%)
Exercise habit^a^ −/+	31/25 (55.4/44.6%)	21/12 (63.6/36.4%)	10/7 (58.8/41.2%)
KLG 2/3/4^a^	52/4/0 (92.9^¶^/7.1/0%)	23/6/4 (69.7/18.2/12.1^¶^%)	8/9/0 (47.1/52.9^¶^/0%)
KOOS Pain^a^	89.2 ± 15.7	84.0 ± 19.5	72.4 ± 25.5^*^
MM_BL_ (kg)	6.24 ± 1.10	6.83 ± 1.27	6.66 ± 1.14
MM_1Y_ (kg)	6.25 ± 1.14	6.75 ± 1.32^‡^	6.35 ± 1.06^‡^
MM_3Y_ (kg)	6.11 ± 1.07^‡,§^	6.63 ± 1.38^‡,§^	6.33 ± 1.02^‡^

In the group (A), the MM_BL_ and MM_1Y_ are significantly higher than the MM_3Y_. In the group (B), the MM_BL_ is higher than the MM_1Y_ and MM_3Y_; the MM_1Y_ is also higher than the MM_3Y_. In the group (C), the MM_BL_ is higher than the MM_1Y_ and MM_3Y_. Cross-sectionally, the mean MM values are not different among three groups on each three occasions. At the BL, the mean KOOS Pain scale is significantly lower in the group (C) than the group (A). The prevalence of KLG 2 is higher in the group (A); that of KLG 4 is higher in the group (B); that of KLG 3 is higher in the group (C) than the other groups.

^a^Value at baseline. Statistical analysis: mixed linear model for continuous value and generalized estimating equations for categorical value: ^*^p ≤ 0.05, compared with group (A); ^†^p ≤ 0.05, compared with group (B); ^¶^p ≤ 0.05, compared with any other group. Repeated measured ANOVA (post-hoc test: Bonferroni): ^‡^p ≤ 0.05, compared with BL; ^§^p ≤ 0.05, compared with 1Y.

BL: baseline; BMI: body mass index; KLG: Kellgren-Lawrence grade; KOOS: Knee injury and osteoarthritis outcome scale; MM: muscle mass; SMML: severe muscle mass loss; 1Y: follow-up after 1 year; 3Y: follow-up after 3 year.

Both crude and adjusted linear regression analysis showed that the effusion area significantly correlated with ΔMM_BL-1Y_ (Table 2). Regarding the ΔMM_1Y-3Y_, the effusion area did not correlate with either the crude or adjusted linear regression model (Table 2). Logistic regression analysis estimated that individuals having ≥30-mm^2^ or 90-mm^2^ suprapatellar effusion at BL showed a significantly higher odds ratio for the prevalence of SMML_BL-1Y_ (Table 2). Conversely, the cut-off values for suprapatellar effusion at BL were not associated with the prevalence of SMML_1Y-3Y_.

**Table 2:** Relationship between the suprapatellar effusion area and the amount of change in the muscle mass of the lower extremity.

Dependent variable	Independent variable	Model	B	P	OR	95% CI		
ΔMM_BL-1Y_ (kg)	Continuous value of Effusion area (mm^2^)	Crude	−0.0015	0.004^*^		−0.0025	—	−0.0005
		Adjusted	−0.0009	0.033^*^		−0.0017	—	−0.0001
ΔMM_1Y-3Y_ (kg)	Continuous value of Effusion area (mm^2^)	Crude	0.0004	0.587		−0.0009	—	0.0016
		Adjusted	−0.0001	0.845		−0.0015	—	0.0013
Prevalence of SMML_BL-1Y_	Cut-off: 30 mm^2^	Crude	1.412	0.047^*^	4.105	1.018	—	16.563
		Adjusted	1.895	0.045^*^	6.653	1.046	—	42.301
	Cut-off: 90 mm^2^	Crude	1.828	0.007^*^	6.222	1.638	—	23.629
		Adjusted	3.071	0.003^*^	21.561	2.779	—	167.265

Statistical analysis: generalized estimating equations, ^*^p ≤ 0.05. Adjusted model is conducted by including the confounding of age, sex, BMI, KOOS pain scale, Kellgren-Lawrence grade, MM, and weekly exercise habit (all at baseline). B: regression coefficient; BL: baseline; MM: muscle mass; OR: odds ratio; P: P-value; SMML: severe muscle mass loss; 95% CI: 95% confident interval.

## Discussion

The current study elucidated the relationship between the quantitative knee effusion value at BL and the degree of longitudinal change in MM of the affected lower extremities in a Japanese population with radiographic-positive KOA. The suprapatellar effusion area evaluated by ultrasonographic imaging was associated with a loss of lower-extremity MM from the BL value to the 1Y-follow-up, whereas this was not evident at the 1Y to 3Y follow-ups. The designated cut-off of 90 mm^2^ showed 21.6-times higher odds for the prevalence of severe muscular atrophy in the affected lower extremity from the BL to the 1Y-follow-up. In the SMML group, the annual rate of MM loss in the lower-extremity was −6.4% during BL to 1Y follow-up, and −6.9% during 1Y to 3Y follow-up on average. Previous studies have reported that middle-aged and older people annually lose muscle mass up to 1% of their total mass on average^33–35^. Therefore, the SMML reported in this study represents a substantial loss. Greater leachate knee effusion was associated with the risk of substantially decreasing the MM in the affected lower extremity, especially in the early phase of diagnosis.

Effusion has drawn research interest due to its harmful effects on the surrounding muscle of the knee joint. The quadriceps muscle, in particular, is the most important muscle surrounding the knee joint. The quadriceps muscle controls knee movement, knee stabilization, and shock absorption^36^. In the past two or three decades, acute and chronic effusion has experimentally been demonstrated to stimulate the knee joint capsule and mechano-receptors, and to inhibit lower motor neuron and quadriceps contraction^13–24^. Accordingly, knee effusion has been reported to alter dynamic muscle function during gait^37^ and jump landing^38^ by diminishing voluntary quadriceps activation^23,38^ and dynamic knee joint power^21^. In line with this experimental evidence, a previous cross-sectional cohort study with KOA subjects clarified that the 3.0-T magnetic resonance imaging findings of effusion were correlated with isokinetic muscle torque weakness in the quadriceps^39^. More recently, Rice *et al*.^40^ performed an intervention study evaluating both aspirating knee effusion and intra-articular steroid injection at the same time for the patients of rheumatoid arthritis, osteoarthritis, and psoriatic arthritis. They measured the peak torque of quadriceps at the pre- and post-intervention levels and found that this invasive intervention immediately regained quadriceps strength^40^. However, a limitation of previous studies focusing on the pathological mechanisms of effusion on the muscle tissue was that the duration of the follow-up period was relatively short. Conversely, it is of note that the current findings have clarified the relatively long-term impairment of knee effusion on the muscle tissue of the affected lower extremity through a three-year follow-up study. In supporting previous evidence, the *in vivo* atrophy of the affected lower extremities could be induced due to the inhibition of muscle contraction in the cohort of patients with radiographic-positive KOA.

Interestingly, in the population of radiographic-positive KOA, the amyotrophic effect of knee effusion on the lower-extremity muscles were altered based on the phase at which we first observed the existence of quantitative levels of leachate effusion using ultrasonography. Knee effusion significantly decreased the MM of lower extremity during the first year from the ultrasonographic examination. Conversely, leachate knee effusion at BL did not influence changes of MM of the lower extremity during the second to third year of the initial examination. Based on the current findings, we speculate that the amyotrophic mechanism of knee effusion on the affected lower extremity could have attenuated based on the follow-up duration from which physicians observed that patients with osteoarthritis presented effusion at their knee joint.

Several limitations of the current study should be emphasized. First, the examination of knee effusion was assessed only at the BL. Therefore, the current study does not clarify the accumulated effect of knee effusion on the muscle atrophy of the affected lower extremity. Consistently, several demographic parameters, such as medication usage, exercise habit, KOOS pain scale, and Kellgren-Lawrence grade, were only available at the BL. In addition, the causes to discharge knee effusion were unclear because we didn’t have the information such as the history of excessive physical activity or knee injury which might affect the development of knee effusion prior to the current ultrasound measurement. Second, the sample sizes of males with massive MM loss were relatively small. Third, knee effusion was evaluated only by a single sagittal ultrasonography image. Multiple images or three-dimensional imaging modalities would clarify the mechanism of knee effusion in more detail. Ideally, evaluation of the relationship between the suprapatellar effusion area and amount of joint aspiration could then be defined. Fourth, BIA could not identify the mass of specific muscles, such as quadriceps, hamstrings, and shank muscles, in the lower extremities. However, BIA has been validated to evaluate the MM by previous studies^28–32^. Finally, the dropout rate of the current subjects was relatively high (58.3%). The sample number of this cohort was depend on the voluntary participation of community residents because this project was performed as part of an annual general health checkup^41^. Therefore, the number of subjects who joined the program for two consecutive years was relatively low. Despite these limitations, the current study clarified the unique mechanism of knee effusion provoking the atrophy of affected lower extremities in individuals having radiographic-positive KOA.

With regard to the clinical relevance of the present study, when physicians diagnose an effusion in patients with KOA, there is a risk of severe muscle atrophy in the affected lower extremity. Controlling knee effusion in KOA patients has the potential to preserve lower-extremity MM by allowing therapeutic effects on the muscle atrophy of lower muscle. Ultrasonography should be useful to evaluate the severity of knee effusion in these patients with KOA. Future studies should address the identification of the mechanism involved in knee effusion on lower-extremity muscle atrophy in the KOA population.

## Conclusions

The quantitative suprapatellar effusion area evaluated by ultrasonography at BL was associated with a loss of MM in the affected lower extremity among a cohort of patients with radiographic-positive KOA. Through the three-year follow-up of our cohort, the harmful effects of knee effusion on decreasing lower-extremity MM were shown to be attenuated comparing the degree of change between the first and the subsequent two-year follow-ups from the BL evaluation. Physicians should pay close attention to any muscle atrophy of patients exhibiting KOA especially when leachate knee effusion is diagnosed during the first year after their examination.

## Data Availability

All data of the current study is available from the database of Department of Orthopaedic Surgery and Social Medicine, Hirosaki University Graduate School of Medicine.
